# Adiponectin Promotes VEGF Expression in Rheumatoid Arthritis Synovial Fibroblasts and Induces Endothelial Progenitor Cell Angiogenesis by Inhibiting miR-106a-5p

**DOI:** 10.3390/cells10102627

**Published:** 2021-10-01

**Authors:** Chien-Chung Huang, Yat-Yin Law, Shan-Chi Liu, Sung-Lin Hu, Jun-An Lin, Chao-Ju Chen, Shih-Wei Wang, Chih-Hsin Tang

**Affiliations:** 1 School of Medicine, China Medical University, Taichung 40402, Taiwan; u104054003@cmu.edu.tw (C.-C.H.); d13437@mail.cmuh.org.tw (S.-L.H.); mqmarkrk@gmail.com (J.-A.L.); monkichi931210@gmail.com (C.-J.C.); 2 Division of Immunology and Rheumatology, Department of Internal Medicine, China Medical University Hospital, Taichung 40402, Taiwan; 3 Institute of Medicine, Chung Shan Medical University, Taichung 40201, Taiwan; andrew.andrewlaw@gmail.com; 4 Department of Orthopedics, Chung Shan Medical University Hospital, Taichung 40201, Taiwan; 5 Department of Medical Education and Research, China Medical University Beigang Hospital, Yunlin 65152, Taiwan; sdsaw.tw@yahoo.com.tw; 6 Department of Family Medicine, China Medical University Hsinchu Hospital, Hsinchu 30210, Taiwan; 7 Department of Medicine, MacKay Medical College, New Taipei City 25245, Taiwan; shihwei@mmc.edu.tw; 8 Graduate Institute of Natural Products, College of Pharmacy, Kaohsiung Medical University, Kaohsiung 80708, Taiwan; 9 Institute of Biomedical Sciences, Mackay Medical College, New Taipei City 25245, Taiwan; 10 Chinese Medicine Research Center, China Medical University, Taichung 40402, Taiwan; 11 Department of Biotechnology, College of Health Science, Asia University, Taichung 40402, Taiwan

**Keywords:** adiponectin, rheumatoid arthritis, angiogenesis, VEGF, miR-106a-5p

## Abstract

Rheumatoid arthritis (RA) is an erosive polyarthritis that can lead to severe joint destruction and painful disability if left untreated. Angiogenesis, a critical pathogenic mechanism in RA, attracts inflammatory leukocytes into the synovium, which promotes production of proinflammatory cytokines and destructive proteases. Adipokines, inflammatory mediators secreted by adipose tissue, also contribute to the pathophysiology of RA. The most abundant serum adipokine is adiponectin, which demonstrates proinflammatory effects in RA, although the mechanisms linking adiponectin and angiogenic manifestations of RA are not well understood. Our investigations with the human MH7A synovial cell line have revealed that adiponectin dose- and time-dependently increases vascular endothelial growth factor (VEGF) expression, stimulating endothelial progenitor cell (EPC) tube formation and migration. These adiponectin-induced angiogenic activities were facilitated by MEK/ERK signaling. In vivo experiments confirmed adiponectin-induced downregulation of microRNA-106a-5p (miR-106a-5p). Inhibiting adiponectin reduced joint swelling, bone destruction, and angiogenic marker expression in collagen-induced arthritis (CIA) mice. Our evidence suggests that targeting adiponectin has therapeutic potential for patients with RA. Clinical investigations are needed.

## 1. Introduction

As a chronic autoimmune condition, rheumatoid arthritis (RA) is marked by symmetric destructive polyarthritis and systemic comorbidities [1]. The attraction of circulating leukocytes into the affected RA joints requires new blood vessels to supply the hypertrophic synovial membrane that is capable of invading the adjacent cartilage and causing bone erosions [2]. Angiogenesis is promoted by proinflammatory cytokines and significant angiogenic factors [3]. The inhibition of synovial angiogenesis is an appealing potential treatment strategy in RA, and avastin, the biologic that targets vascular endothelial growth factor (VEGF), has demonstrated therapeutic effects in type II collagen-induced arthritis (CIA) [4].

Adiponectin, the most abundant of all adipokines, is secreted by adipocyte tissue [5]. Adiponectin activates the AMPK and PPAR-α signaling pathways to regulate glucose and fatty acid metabolism [6]. It is also becoming clear that adiponectin is an important contributor to chronic inflammation, as is seen in cardiovascular disease, osteoarthritis (OA), the metabolic syndrome, and also RA [7]. Elevated levels of adiponectin have been found in human samples of RA serum and synovial fluid [8,9], while other research has determined that it is possible to predict radiographic joint damage from baseline serum adiponectin values in RA patients, and it is also established that adiponectin stimulates the production of interleukin (IL)-6, prostaglandin E2, and MMP in RA synovial fibroblasts [10,11]. However, the effect of adiponectin on RA angiogenesis is unclear. Adiponectin reportedly induces the production of VEGF in RA synovial fibroblasts and osteoblasts [12,13,14], and increases the expression of the inflammatory indicator endocan in RA synovial fibroblasts [15]. Cultured circulating endothelial progenitor cells (EPCs) can enhance blood vessel formation and have been used to induce angiogenesis and vascular repair under experimental conditions [16,17]. Previous reports have described elevated levels of EPCs in the RA synovial tissue [18], while enhanced late-outgrowth circulating EPC levels correlate positively with RA severity [19]. Research is needed to determine how exactly adiponectin mediates EPC-dependent angiogenesis in RA. 

The short noncoding RNAs, microRNAs (miRNAs), post-transcriptionally modulate gene manifestations [20]. Various miRNA genes expressed in immune, inflammatory, and synovial cells from patients with RA [21] can cause synovial hyperplasia and bone damage, or promote inflammation, through positive or negative manipulation [22]. MiRNAs play crucial roles in adiponectin-associated metabolic syndrome, diabetes mellitus, fatty liver, and several cancers [23,24,25,26]. However, evidence is lacking as to miRNA activity during adiponectin treatment in RA. Our research has shown that adiponectin stimulates VEGF-dependent angiogenesis in RA synovial fibroblasts via MEK/ERK signaling and by downregulating miRNA-106a-5p (miR-106a-5p) expression. Inhibition of adiponectin significantly mitigated paw swelling, erosion of bone, and angiogenesis in the CIA mouse model. Taken together, the results help to clarify how adiponectin enhances angiogenic activity in inflamed joints of RA and suggest that an anti-angiogenic strategy targeting adiponectin would be beneficial for this disease.

## 2. Materials and Methods

### 2.1. Cell Culture

The MH7A cell line (human RA synovial fibroblasts) was obtained from Riken (Ibaraki, Japan) and the cell culture conditions were maintained according to established procedures [27,28]. Experiments were performed using 5 × 10^6^ cells from passages 3 to 9.

Human endothelial progenitor cells (EPCs) were prepared according to our previous protocols [29,30], after we obtained approval from the Institutional Review Board (IRB) of Mackay Medical College, New Taipei City, Taiwan (reference number: P1000002). Peripheral blood (80 mL) was collected from healthy donors after they completed written informed consent forms. Mononuclear cells (3 × 10^7^ cells) were isolated from blood components by centrifugation on Ficoll-Paque PLUS (Amersham Biosciences, Uppala, Sweden). EPCs were maintained and characterized as follows: briefly, EPCs were seeded on gelatin-coated dishes containing MV2 medium, SupplementMix (PromoCell, Heidelberg, Germany), and 20% nonheat-inactivated defined fetal bovine serum (FBS; HyClone, Logan, UT, USA). EPCs were characterized with CD34^+^/CD133^+^/VEGFR2^+^ antibodies using a FACSCalibur flowcytometer and CellQuest software (BD Biosciences, San Jose, CA, USA) [31].

### 2.2. qRT-PCR Gene Expression Analysis of mRNA and miRNA

TRIzol reagent (Invitrogen, Waltham, MA, USA) was used to extract MH7A RNA. Subsequently, miRNA was detected according to the manufacturer’s instructions of the Mir-XTM miRNA First Strand Synthesis Kit (Applied Biosystems, Foster City, CA, USA). We performed qPCR analysis according to an established protocol [32,33].

### 2.3. Western Blot Analysis

MH7A cells (5 × 10^5^ cells) were seeded into 6-well plates. Cell lysate was collected and separated as previously described [34,35]. All specific primary antibodies: anti-VEGF antibody (A17877; Abclonal, MA, USA), β-actin (SC-47778), p-MEK (SC-271914), MEK (SC-6250), p-ERK (SC-7383), and ERK (SC-1647) antibodies (Santa Cruz biotechnology, Dallas, TX, USA) were used for 24 h. The densities of specific bands were visualized by chemiluminescence (ECL) reagents (WBKLS0500, Millipore Corp., Billerica, MA, USA).

### 2.4. Enzyme-Linked Immunosorbent Assay (ELISA)

The specific VEGF-A ELISA kits (DY293B; R&D, Minneapolis, MN, USA) were used to measure the VEGF levels in conditioned medium. MH7A cells were transfected with specific adiponectin shRNA plasmids (National RNAi Core Facility, Taipei, Taiwan) and respective siRNAs (Dharmacon, Lafayette, CO, USA), or treated with specific inhibitors of PD98059 (P215) and U0126 (U120) (Sigma-Aldrich, St. Louis, MO, USA), then incubated with adiponectin. The conditioned medium was collected according to the manufacturer’s instructions [36].

### 2.5. EPC Tube Formation

Tube formation was analyzed, as previously described [37]. Matrigel (BD Biosciences, Bedford, MA, USA) was coated onto 48-well plates and EPCs (2 × 10^4^ per 100 μL) were resuspended in MV2 serum-free medium with the indicated adiponectin concentration for 24 h, then added to the wells. After 12 h of incubation at 37 °C, EPC tube formation was assessed with a photomicroscope, and each well was photographed at 200× magnification. EPC tube formation was subjected to quantitative analysis software (ImageJ softwell). 

### 2.6. Transwell Migration Assay

The Transwell migration assay was conducted, as previously described [37]. The number of cells per field of view was calculated using a Nikon ECLIPSE TS100 imaging optical microscope.

### 2.7. Plasmid Construction and Luciferase Assay

The wild type VEGF 3′-UTRs with miR-106a-5p binding sites were inserted into the pmirGLO luciferase reporter vector (Promega, Madison, WI, USA). The mutant VEGF 3′UTR region (MT-VEGF-3′-UTR) was obtained from Invitrogen (Waltham, MA, USA). Finally, luciferase activity was determined using a dual-luciferase reporter assay system (Promega, Madison, WI, USA).

### 2.8. The Chick Chorioallantoic Membrane (CAM) Assay

The CAM assay evaluated in vivo angiogenic activity, as previously described [38]. Angiogenesis activities were detected by microscopy and digital photographs.

### 2.9. In Vivo Matrigel Plug Assay

The protocol was performed as previously described [39]. Immunofluorescent staining was performed using anti-VEGF, anti-CD31, anti-CD34 (Abcam, Cambridge, MA, USA), and anti-CD133 (Biorbyt, Cambridge, MA, USA). 

### 2.10. Collagen-Induced Arthritis Mouse Model

The emulsion contained bovine type II collagen (CII, Chondrex, Redmond, WA, USA) and IFA Freund’s incomplete adjuvant (Sigma-Aldrich. St. Louis, MO, USA) intradermally injected into each mouse tail root on day 0, and we intra-articularly injected the same amount at day 14, according to the approved method [40]. 

Arthritis in CIA mice reliably develops within 6 weeks, and more than 90% of the mice will develop severe arthritis. Following both immunizations, the mice were allocated to the Control (*n* = 8), CIA (*n* = 8), and adiponectin shRNA (*n* = 8) groups. The mice received intra-articular injections with lentiviral adiponectin shRNA and sacrificed on day 56 of treatment. Paw swelling was measured in a blinded manner with a plethysmometer (Marsap, Mumbai, India) once weekly for 4 weeks to determine the clinical severity of arthritis. The tissues between ankle and phalangeal joints were prepared for micro-computed tomography (micro-CT) examinations.

### 2.11. Ethics Statement

All experiments involving human clinical samples were approved by the Institutional Review Board of China Medical University Hospital, which granted approval for this study to proceed (Approval no. CMUH108-REC3-039). All experiments involving animals were conducted according to the ethical policies and procedures issued by the Institutional Animal Care and Use Committee of China Medical University, which approved our experiments (Approval no. 2018-102).

### 2.12. Statistical Analysis

All results were analyzed using GraphPad Prism version 5.0 (GraphPad Software, Inc., La Jolla, CA, USA) and all values are expressed as the mean ± S.D. Differences between selected pairs from the experimental groups were analyzed for statistical significance by the paired sample *t*-test. The statistical difference was regarded as significant when the *p* value was <0.05.

## 3. Results

### 3.1. Adiponectin Increases VEGF-Dependent EPC Angiogenesis in MH7A Cells

**Figure 1:**
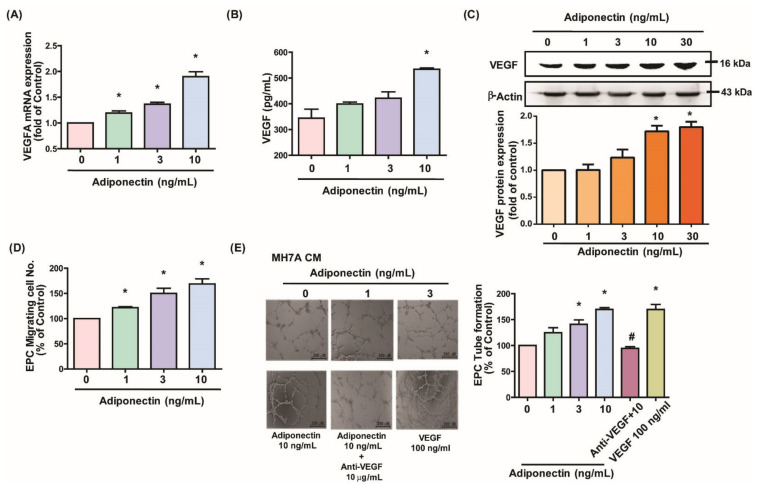
Adiponectin stimulates VEGF expression in MH7A cells and promotes EPC angiogenesis. (**A**–**C**) MH7A cells were treated with prespecified concentrations of adiponectin (0–10 ng/mL) for 24 h. VEGF expression was examined by qPCR, ELISA, and Western blotting (*n* = 4 per group). (**D**,**E**) MH7A cells were treated with adiponectin (0–10 ng/mL) or pretreated with 30 min with VEGF antibody for 30 min then stimulated with adiponectin (10 ng/mL) for 24 h. Medium was collected as CM; 200 μL of 20% FBS MV2 medium and 150 μL of MH7A CM was applied to the EPCs. Capillary-like structure formation and in vitro cell migration in EPCs were examined by the Transwell and tube formation assays. Direct application of VEGF (100 ng/mL) served as the positive control in EPC tube formation and migration (*n* = 5 per group). * *p* < 0.05 versus the control group; ^#^ *p* < 0.05 versus the adiponectin-treated group.

**Figure 2:**
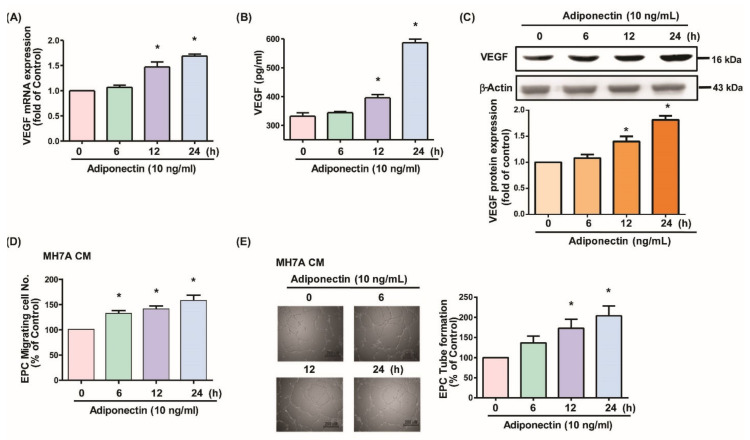
Adiponectin time-dependently enhances VEGF production in MH7A cells and EPC angiogenesis. (**A**–**C**) MH7A cells were treated with adiponectin (10 ng/mL) for 6, 12, and 24 h. VEGF expression was examined by qPCR, ELISA, and Western blotting (*n* = 4 per group). (**D**,**E**) MH7A cells were treated with adiponectin (10 ng/mL) for 6, 12, and 24 h. Medium was collected as CM; 200 μL of 20% FBS MV2 medium and 150 μL of MH7A CM was applied to the EPCs. Capillary-like structure formation and in vitro cell migration in EPCs were examined by the Transwell and tube formation assays (*n* = 5 per group). * *p* < 0.05 versus the control group.

First, to understand the association between adiponectin and EPCs in RA, we examined whether adiponectin stimulates EPC functions in MH7A cells. Adiponectin treatment of MH7A cells concentration- and time-dependently upregulated VEGF mRNA and protein expression (Figure 1A–C and Figure 2A–C). Incubating EPCs in culture medium from adiponectin-treated synovial fibroblasts concentration- and time-dependently upregulated EPC tube formation and migration activities. Adding VEGF monoclonal antibody decreased adiponectin-dependent tube formation and migration activities (Figure 1D,E and Figure 2D,E). These results indicate that adiponectin induces EPC angiogenesis via VEGF upregulation in MH7A cells.

### 3.2. MEK/ERK Signaling Is Critical for Adiponectin-Dependent EPC Angiogenesis

**Figure 3:**
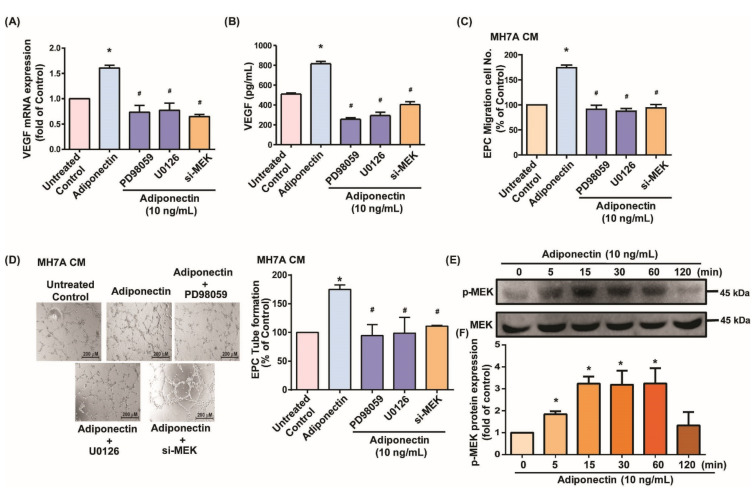
The MEK signaling pathway is required in adiponectin-induced EPC migration and tube formation. (**A**,**B**) MH7A cells were left untreated, stimulated with adiponectin (10 ng/mL) alone for 24 h, or pretreated with MEK inhibitors (PD98059 and U0126) or transfected with MEK siRNA for 30 min, prior to 24 h of adiponectin (10 ng/mL) stimulation. VEGF expression was examined by qPCR and ELISA assays (*n* = 4 per group). (**C**,**D**) Collected CM from MH7A was applied to EPCs and angiogenesis was examined by the Transwell and tube formation assays (*n* = 5 per group). (**E**) MH7A cells were treated with adiponectin for varying amounts of time and Western blot (*n* = 3) determined MEK phosphorylation. (**F**) Densitometry analysis of (**E**). * *p* < 0.05 versus the control group; ^#^ *p* < 0.05 versus the adiponectin-treated group.

**Figure 4:**
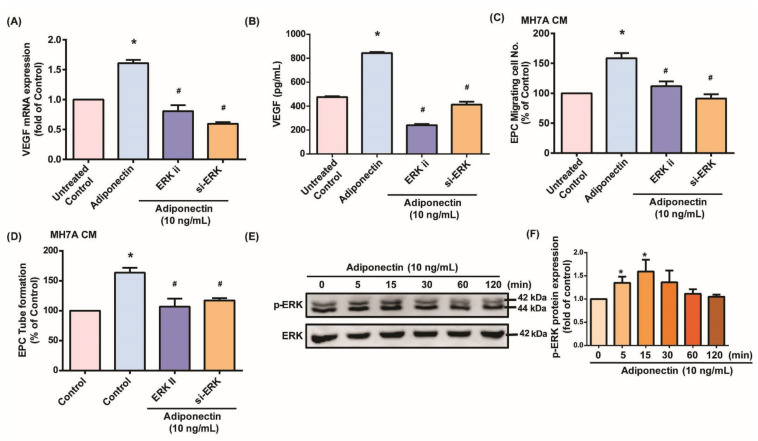
The ERK signaling pathway is essential in adiponectin-stimulated EPC migration and tube formation. (**A**,**B**) MH7A cells were left untreated, stimulated with adiponectin (10 ng/mL) alone for 24 h, or pretreated with ERK inhibitors (ERK II) or transfected with ERK siRNA for 30 min, prior to 24 h of adiponectin (10 ng/mL) stimulation. VEGF expression was examined by qPCR and ELISA assays (*n* = 4 per group). (**C**,**D**) Collected CM from MH7A was applied to EPCs and angiogenesis was examined by the Transwell and tube formation assays (*n* = 5 per group). (**E**) MH7A cells were treated with adiponectin for varying amounts of time and Western blot (*n* = 3) determined ERK phosphorylation. (**F**) Densitometry analysis of (**E**). * *p* < 0.05 versus the control group; ^#^ *p* < 0.05 versus the adiponectin-treated group.

The MEK/ERK signaling pathway has been regarded as important in adiponectin regulating cell functions [41,42,43]. We therefore checked the involvement of MEK/ERK signaling in adiponectin-enhanced VEGF production and angiogenesis. Incubating MH7A cells with adiponectin time-dependently increased MEK and ERK phosphorylation (Figure 3E and Figure 4E). We then pretreated MH7A cells with MEK inhibitors (PD98059, U0126), an ERK inhibitor (ERK II), or transfected the cells with MEK or ERK siRNAs. The results showed that both MEK/ERK inhibitors and siRNAs reduce adiponectin-induced VEGF secretion (Figure 3A,B and Figure 4A,B). The effects of adiponectin on EPC angiogenic activities were also abolished (Figure 3C,D and Figure 4C,D). Thus, adiponectin enhances VEGF expression and EPC angiogenesis in MH7A cells via MEK/ERK signaling.

### 3.3. Adiponectin Enhanced VEGF-Dependent Angiogenesis via Downregulation of Adiponectin-Mediated miR-106a-5p Expression

**Figure 5:**
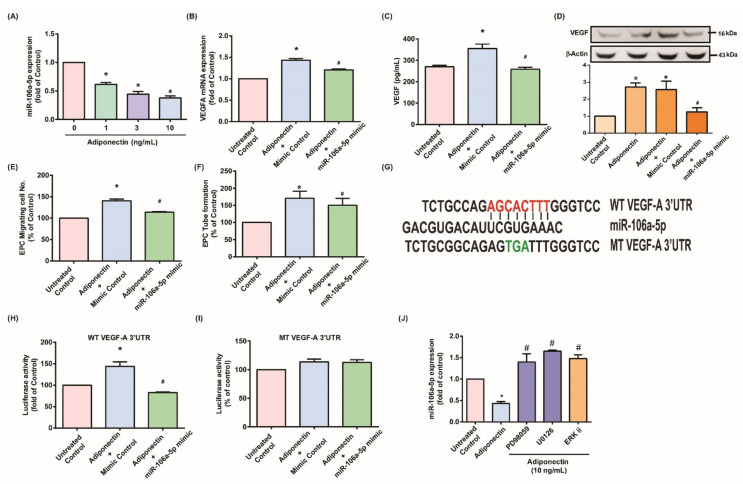
Adiponectin enhances VEGF-dependent angiogenesis via the suppression of miR-106a-5p. (**A**) MH7A cells were incubated with various concentrations adiponectin for 24 h. miR-106a-5p expression was determined by the qPCR assay. (**B**–**D**) MH7A cells were transfected with mimic control or miR-106a-5p mimic for 24 h, then stimulated with adiponectin for 24 h. VEGF levels were determined by qPCR, ELISA, and Western blot assay (*n* = 4). (**E**,**F**) Collected CM was applied to EPCs and EPC angiogenesis was quantified. (**G**) Schematic 3′UTR representation of human VEGF containing the miR-106a-5p binding site. (**H**,**I**) MH7A cells were transfected with the indicated luciferase plasmid with or without miR-106a-5p mimic for 24 h, then stimulated with adiponectin for 24 h. Relative luciferase activity was determined. (**J**) MH7A cells were pretreated with MEK or ERK inhibitors for 30 min, then stimulated with adiponectin for 24 h. miR-106a-5p expression was quantified by qPCR. * *p* < 0.05 versus the control group; ^#^ *p* < 0.05 versus the adiponectin-treated group.

Associations between miRNA expression and RA pathogenesis have been mentioned previously [44], although how miRNAs might mediate RA disease during adiponectin administration is unclear. Our analysis of the miRNA prediction algorithms RNAhybrid, miRmap, miRWalk, and TargetScan identified miR-106a-5p as a candidate miRNA capable of binding to the VEGF 3′-UTR region. Adiponectin treatment of MH7A cells caused concentration-dependent decreases in miR-106a-5p expression (Figure 5A). Transfecting the cells with the miR-106a-5p mimic effectively mitigated adiponectin-induced VEGF production (Figure 5B–D), EPC tube growth, and EPC migratory activities (Figure 5E,F). WT-and MT-VEGF 3′-UTR luciferase plasmids were constructed to check whether adiponectin mediated effects decrease the targeting of miR-106a-5p to VEGF 3′-UTRs (Figure 5G). Whereas WT-VEGF 3′-UTR plasmids displayed adiponectin-induced increases in luciferase activity, this phenomenon did not occur in MT-VEGF 3′-UTRs (Figure 5H,I). MiR-106a-5p mimic reversed adiponectin-stimulated luciferase activity (Figure 5H,I), and incubation with MEK and ERK inhibitors blocked adiponectin from downregulating miR-106a-5p (Figure 5J). These data suggest that adiponectin stimulates angiogenic factor production by lowering miR-106a-5p levels via the MEK and ERK signaling cascades. 

### 3.4. Suppressing Adiponectin Reduces RA Angiogenesis

**Figure 6:**
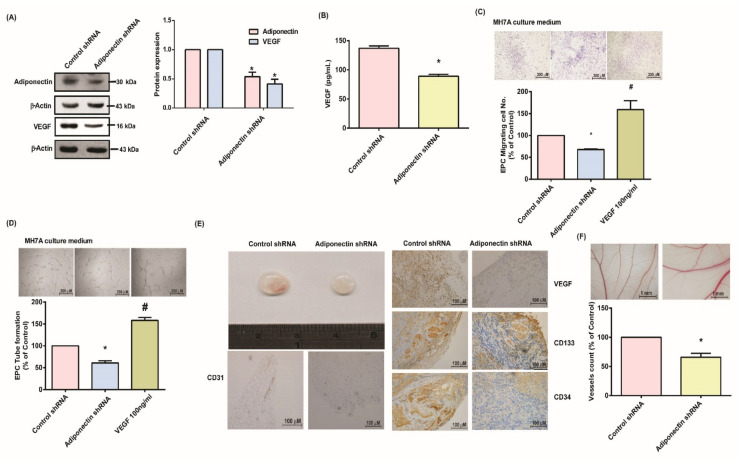
Blocking adiponectin attenuates RA angiogenesis in vivo. (**A**,**B**) MH7A cells were infected with adiponectin shRNA for 24 h. Adiponectin and VEGF expression was examined by Western blot (*n* = 4) and ELISA (*n* = 4). (**C**,**D**) Collected CM was applied to EPCs and EPC angiogenesis was quantified. (**E**) Matrigel plugs containing the harvested CM were subcutaneously injected into the flanks of nude mice. After 7 days, the plugs were photographed and hemoglobin levels were quantified. Plug specimens were immunostained with CD31 (*n* = 6), CD34 (*n* = 6), and CD133 (*n* = 6) antibodies. (**F**) After subjecting MH7A cells to the treatment conditions as indicated, the harvested CM was applied to 5-day-old fertilized chick embryos for 3 days. CAMs were examined by microscopy and photographed (*n* = 6), and vessels were counted manually. * *p* < 0.05 versus the control group; ^#^ *p* < 0.05 versus the adiponectin shRNA-transfected group.

In vivo CAM and Matrigel plug experiments examined the angiogenic effects of adiponectin in RA disease. When we transfected adiponectin shRNA into MH7A cells, we observed decreases in adiponectin and VEGF expression (Figure 6A,B). Transfection of the cells with adiponectin shRNA downregulated EPC tube formation and migration, which was reversed by the addition of VEGF (Figure 6C,D). Knockdown of adiponectin attenuated microvasculature development in Matrigel plugs, evaluated by staining with VEGF, CD31, CD34, and CD133 (Figure 6E). Similarly, blood vessel formation in the CAM assay was suppressed by adiponectin shRNA (Figure 6F). These preclinical data confirm that suppression of adiponectin reduces angiogenesis in RA synovial fibroblasts. 

### 3.5. Attenuating Bone Damage and Angiogenesis by Targeting Adiponectin in CIA Mice

**Figure 7:**
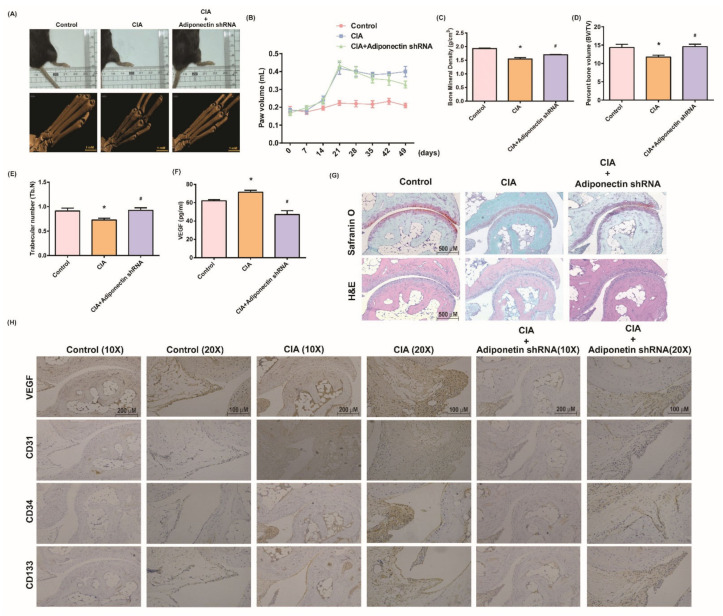
Lentivirus carrying adiponectin short hairpin RNA (sh-adiponectin) reduces bone erosion and angiogenic expression in a CIA model. (**A**,**B**) CIA mice received intra-articular injections of 7.1 × 10^6^ PFU adiponectin shRNA on day 14 and were euthanized on day 49. Hind paw swelling was photographed and measured with a digital plethysmometer in the different groups (Control, CIA, and CIA mice receiving intra-articular lentiviral sh-adiponectin; *n* = 8 per group). Representative micro-CT images of the hind paws were recorded on Day 56. (**C**–**F**) Micro-CT SkyScan Software quantified bone mineral density (BMD), bone volume percentage (BV/TV), and trabecular numbers (Tb. N.). VEGF serum levels were determined by ELISA. (**G**,**H**) Histological sections of ankle joints were stained with H&E or Safranin O and immunostained with CD31, CD34, and CD133. * *p* < 0.05 versus the control group; ^#^ *p* < 0.05 versus the untreated CIA group.

We sought to determine whether blocking adiponectin would attenuate destructive synovitis in CIA mice. Lentiviral adiponectin shRNA injections significantly reduced hind paw swelling in CIA mice compared with control mice (Figure 7A,B). Micro-CT imaging revealed worse bone erosion of hind paws in CIA mice without additional treatment, whereas the mice infected with lentiviral adiponectin shRNA had less bone damage with higher bone mineral density, bone volume, and trabecular numbers (Figure 7A,C–E). Serum VEGF expression was also decreased in adiponectin shRNA-treated CIA mice (Figure 7F). Intra-articular cartilage damage according to pathologic examination (Figure 7G) revealed significantly increased expression of EPC and angiogenesis vessel markers in the CIA mice, versus significant reductions in the adiponectin shRNA treatment group (Figure 7H). Thus, lentiviral adiponectin shRNA administration appears to protect against bone damage and reduce angiogenesis in an RA animal model. 

## 4. Discussion

RA synovial fibroblasts secrete various proinflammatory cytokines that contribute to surrounding cartilage and bone damage [45]. During the development of RA disease, angiogenesis facilitates oxygen and nutrient transportation to B cells, T cells, or macrophages in the inflamed site and propagates the inflamed synovium with immune cell infiltration [3]. RA clinical studies using musculoskeletal ultrasound have shown that subclinical synovitis detected by power Doppler sonography is associated with bone damage [46] and that sonographic signals of hypervascularity correlate with angiogenic VEGF levels [47]. Thus, inhibiting neovascularization might further ameliorate RA severity in treatment-refractory patients [48]. We are the first research group to describe how adiponectin promotes angiogenic activities in RA via MEK/ERK signaling and by downregulating miR-106a-5p. Knockdown of adiponectin appears to attenuate synovitis severity and destruction of bone in CIA animal experiments. 

Adipokines act as biologically active substances in neuroendocrine–immune interactions. Adipokine synthesis in the joint microenvironment can occur through the activities of synoviocytes, osteoblasts and osteoclasts, chondrocytes, and inflammatory cells [49]. Most of these adipokines, including adiponectin, visfatin, resistin, and leptin, display proinflammatory effects in rheumatic joint disorders. Adiponectin plasma levels positively correlate with RA disease activity [8,9,50]. Adiponectin stimulates the expression of various proinflammatory cytokines in RA synovial fibroblasts [51], although the effects of adiponectin on EPC angiogenesis in RA have not been reported previously. It is established that adiponectin increases VEGF secretion in RA synovial fibroblasts and osteoblasts [12,13,14] and upregulates the expression of endocan, an angiogenic proteoglycan, in synovial fibroblasts [15,52]. Our data detail how adiponectin increases VEGF production in RA synovial fibroblasts and EPC angiogenesis via intracellular signal pathways. 

Various proangiogenic factors, including VEGF, fibroblast growth factor, and PDGF, are involved in the angiogenic processes of several different diseases, including arthritis [53], and may interfere with the basal levels of EPC tube formation. Incubation of MH7A cells with adiponectin concentration-dependently promotes VEGF synthesis, resulting in EPC angiogenesis. Interestingly, we found that VEGF antibody significantly antagonized increases in EPC angiogenesis induced by adiponectin, when we stimulated MH7A cells for 24 h in CM. This suggests that VEGF is a vital modulator in EPC-mediated angiogenesis during RA development.

RA angiogenesis is driven by proinflammatory cytokines and proangiogenic mediators released from adjacent tissues or the systemic circulation [3]. Recent observations report that CCL28 and CCR10 expression is upregulated in RA synovial tissue and synovial fluid and that RA angiogenesis is promoted by joint CCL28, which activates the ERK pathway [54]. Another study has revealed that silencing of *granzyme B* gene expression protects against RA articular injury by suppressing inflammatory and angiogenic factors (VEGF and basic fibroblast growth factor) through the MEK/ERK signaling pathway [55]. Our results indicate that adiponectin induces VEGF-dependent angiogenesis in RA synovial fibroblasts via the MEK/ERK intracellular pathway. 

MiRNAs appear to be pivotal regulators of biological functions and pathological illnesses [56]. Altered miRNA expression leads to immune dysregulation, inflammation, or cellular proliferation in RA [54]. In particular, miR-106a-5p participates in the proliferative, invasive, and metastatic behaviors of numerous cancers [57,58,59]. One OA study has revealed that cryptotanshinone, a *Salvia miltiorrhiza* Bunge root extract, protects cartilage against degeneration via the miR-106a-5p/GLI-similar 3 (GLIS3) axis [60]. MiR-106a-5p expression was downregulated in human OA cartilage, whereas GLIS3 expression was upregulated [60]. In another study, long non-coding RNA H19 regulated proliferation and apoptosis of OA chondrocytes by modulating miR-106a-5p expression [61]. In our study, adiponectin promoted EPC tube formation and migration by suppressing miR-106a-5p expression in RA. 

In CIA animal models, intra-articular adiponectin administration attenuates arthritis by downregulating IL-1, MMP-3, and TNF-α expression in inflamed joints [62,63]. Conversely, other studies demonstrate that adiponectin exacerbates CIA by promoting Th17 and receptor activator of nuclear factor-κB ligand (RANKL) expression [64], or by upregulating osteopontin [65]. Furthermore, although systemic delivery of adiponectin by adenovirus vectors can mitigate CIA disease [66], rheumatic symptoms in CIA were markedly attenuated by administration of monoclonal antibodies against adiponectin isomers [67]. Thus, adiponectin may be characterized by proinflammatory or anti-inflammatory effects in RA, depending on the isoforms, exposure time, concentrations, or the microenvironment [51,68]. 

## 5. Conclusions

**Figure 8:**
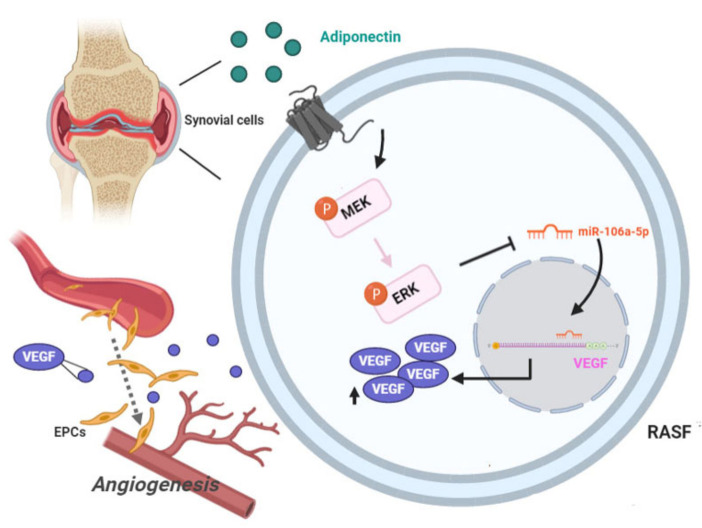
The schematic diagram summarizes the mechanism whereby adiponectin upregulates VEGF expression in MH7A cells. Adiponectin induces VEGF expression and angiogenesis through the MEK/ERK signaling cascade and reduces miR-106-5p expression in MH7A cells.

More investigation is called for to clarify the interactions between adiponectin and RA. Our data prove that a link exists between adiponectin and EPC angiogenic activities in MH7A cells. This process is VEGF-dependent and regulated by MEK/ERK and miR-106a-5p. Our CIA mouse experiments demonstrate that blocking adiponectin significantly ameliorates RA severity. Thus, targeting adiponectin holds promise in the treatment of RA and deserves to be investigated clinically (Figure 8). 

## Data Availability

The data presented in this study are available on request from the corresponding author.
